# Control of the Nitriding Process of AISI 52100 Steel in the NH_3_/N_2_ Atmosphere

**DOI:** 10.3390/ma18133041

**Published:** 2025-06-26

**Authors:** Jerzy Michalski, Tadeusz Frączek, Rafał Prusak, Agata Dudek, Magdalena Kowalewska-Groszkowska, Maciej Major

**Affiliations:** 1 Łukasiewicz Research Network—Warsaw Institute of Technology, 01-796 Warszawa, Poland; jerzymichalski987@gmail.com; 2 Department of Materials Engineering, Faculty of Production Engineering and Materials Technology, Czestochowa University of Technology, 42-201 Czestochowa, Poland; tadeusz.fraczek@pcz.pl (T.F.); agata.dudek@pcz.pl (A.D.); 3 Department of Production, Faculty of Production Engineering and Materials Technology, Czestochowa University of Technology, 42-201 Czestochowa, Poland; 4 Museum and Institute of Zoology of the Polish Academy of Sciences, 05-152 Czosnów, Poland; mkowalewska@miiz.waw.pl; 5 Faculty of Civil Engineering, Czestochowa University of Technology, 42-201 Czestochowa, Poland; maciej.major@pcz.pl

**Keywords:** registration of mass change, gas nitriding, nitrogen availability, nitrogen potential, nitrogen-diluted atmosphere

## Abstract

This paper proposes a mathematical description of nitriding atmospheres obtained from a one-component ammonia ingoing atmosphere and a two-component ammonia inlet nitrogen-diluted atmosphere. The Fe-N phase equilibrium diagrams of the nitriding atmosphere in the hydrogen content-temperature (Q-T) system for selected NH_3_/N_2_ atmosphere compositions are presented. The nitriding atmosphere obtained with different degrees of nitrogen dilution of the ingoing atmosphere was characterized. It has been shown that in processes carried out in nitriding atmospheres obtained from a two-component atmosphere with nitrogen, there is no direct relationship between the value of the nitrogen potential and the degree of dilution of the ingoing atmosphere with nitrogen. It has been shown analytically and confirmed experimentally that with changes in the degree of dilution of ammonia with nitrogen, the hydrogen content of the nitriding atmosphere and, consequently, the nitrogen availability of the nitriding atmosphere change. Using the example of nitriding AISI 52100 steel, it has been experimentally demonstrated that the change in nitrogen availability, caused by a change in the degree of dilution of the ingoing atmosphere with nitrogen, is not accompanied by a change in the value of the nitrogen potential. It has also been shown that the change in the nitrogen availability of the nitriding atmosphere, induced by the change in the composition of the *a*NH_3_/*b*N_2_ ingoing atmosphere, affects the kinetics of nitrogen mass gain in the nitrided layer and the distribution of nitrogen mass between the iron nitride layer and the solution zone. It has also been shown that with the change in nitrogen availability, what changes in addition to the thickness of the iron nitride layer is also the phase composition of the layer. Using gravimetric tests, the mass of nitrogen in the iron nitride layer and the solution zone has been determined. To describe the equilibrium between the NH_3_/H_2_ atmosphere and nitrogen in the different iron phases, a modified Lehrer diagram in the coordinate system of temperature and hydrogen content in the nitriding atmospheres (T-Q) has been proposed.

## 1. Introduction

The kinetics of the increase in thickness of the nitrided layer, as well as the phase composition of the iron nitride layer, is a function of the surface nitrogen concentration. In controlled gas nitriding processes in a nitriding atmosphere obtained from a two-component ingoing atmosphere with hydrogen (NH_3_/H_2_), the nitrogen surface concentration is a function of the nitrogen potential [1]. In processes carried out in a nitriding atmosphere obtained from a two-component ingoing atmosphere (outgoing atmosphere) with nitrogen (NH_3_/H_2_), the surface concentration of nitrogen, at a constant ingoing atmosphere flow rate, depends on the degree of dilution of the ingoing atmosphere with nitrogen [2]. In depressurized processes, the surface concentration of nitrogen at a constant flow rate is a function of pressure [3]. Consequently, regardless of the process, the surface nitrogen concentration is determined by the relationship between the nitrogen flux from the atmosphere to the surface and the nitrogen flux into the nitrided steel. A quantitative measure of the first stream is the nitrogen availability of the nitriding atmosphere, which is a function of the degree of ammonia/hydrogen dissociation in the nitriding atmosphere and the flow rate of the ingoing atmosphere [4]. The magnitude of the second parameter depends on the properties of the nitrided steel, in particular on the content of nitride-forming elements [5]. With an increase in nitrogen availability of the nitriding atmosphere, the surface concentration of nitrogen increases, while an increase in the content of nitride-forming alloying elements in the substrate, in combination with carbon, has the opposite effect [2].

The ammonia atmosphere is the reference atmosphere for all types of nitriding atmospheres used in nitriding processes. During the nitriding process, ammonia is partially transformed into a mixture of nitrogen and hydrogen. The degree of ammonia transformation depends on the temperature, the flow rate of the ingoing atmosphere, and the size of the surface area of the nitrided charge. At a constant temperature and flow rate in the furnace retort, the degree of conversion of ammonia into a mixture of nitrogen and hydrogen is determined, which is expressed by the nitrogen potential. For the NH_3_ ingoing atmosphere, each value of nitrogen potential corresponds to the same volume of hydrogen released during the ammonia dissociation reaction. If the ingoing atmosphere is a mixture of ammonia and nitrogen NH_3_/N_2_, the volume of hydrogen released as a result of ammonia dissociation will change with the change in the degree of dilution of ammonia with nitrogen in the ingoing atmosphere.

This paper proposes a description of nitriding atmospheres obtained from a one-component ammonia inlet atmosphere (NH_3_) and a two-component inlet atmosphere (NH_3_/N_2_). The Fe-N phase equilibrium diagrams of the outgoing atmosphere in the hydrogen content-temperature (Q-T) system for selected NH_3_/N_2_ atmosphere compositions are presented.

In the processes of nitriding AISI 52100 steel, studies have been carried out to determine to what extent the change in the nitrogen availability of the nitriding atmosphere will affect the kinetics of nitrogen mass gain in the nitrided layer and the distribution of nitrogen mass between the iron nitride layer and the solution zone. The purpose of the experiments has also been to verify whether the change in nitrogen availability changes the phase composition of the layer, in addition to the thickness of the iron nitride layer.

## 2. Characteristics of the Nitriding Methods Used

The varieties of nitriding in thermochemical treatment used in practice are mainly gas nitriding at atmospheric pressure [1,6,7,8], fluorescent nitriding [9,10,11], laser nitriding [12], reduced-pressure nitriding [13,14,15], fading fluidized nitriding [16,17] as well as nitriding, or actually bath carbonitriding. Among the gas varieties at atmospheric pressure, regulated gas nitriding by NITREG (RAG) [18,19,20,21] and, in recent years, regulated nitriding by ZeroFlow [22,23,24] and reduced pressure nitriding (LPN) [13,25] are commonly used. The control of the nitriding process, both in the NITREG and ZeroFlow processes, consists of regulating the nitrogen potential, the value of which determines the nitrogen concentration on the surface of the nitrided steel and, consequently, the phase composition of the near-surface layer of iron nitrides [26]. The phase composition of the nitrides, in turn, affects the thickness of the solution zone of the nitrided layer. In these processes, one value of nitrogen potential corresponds to one value of nitrogen availability of the nitriding atmosphere; any change in nitrogen potential results in a change in nitrogen availability. Ratajski [27] showed that if during the nitriding process the nitrogen potential takes values corresponding to the persistence of the ε phase, thicker solution zones of the nitrided layer are obtained than when the nitrogen potential takes values corresponding to the persistence of the γ phase. The slowdown in growth kinetics of the solution zone is due to the greater homogeneity of the phase γ’. Therefore, it is advantageous to keep the value of the nitrogen potential in the region of phase persistence ε, which guarantees maximum growth kinetics of the solution zone at a given process temperature. On the other hand, under such conditions, a thick layer of iron nitrides is formed, which is often undesirable. Limiting the kinetics of iron nitride thickness growth requires lowering the nitrogen potential, which limits the growth kinetics of the solution layer.

The nitriding atmosphere that allows the flux of nitrogen to the surface of the nitrided steel to change at a constant nitrogen potential is the nitriding atmosphere obtained from the two-component ingoing atmosphere NH_3_/N_2_. Smirnov and Kuleshov [28] carried out thermodynamic calculations of the reaction of iron nitride formation under different conditions of nitrogen-dilution of the ingoing atmosphere and argon for atmospheric pressure and reduced pressure. They calculated the limiting degrees of dissociation for the interfacial boundaries α/γ’ and γ’ε and showed equilibrium diagrams between the solid phases of the Fe-N system and the ammonia nitrogen gas mixture for different nitrogen dilutions. The authors noted that only about 0.64% of the nitrogen that forms during the dissociation of ammonia is used for building the nitrided layer.

In his monograph [5], Michalski presented characterizations, calculations, and analytical formulas to design and simulate nitrogen-diluted ingoing atmospheres to achieve dissociation limits for interfacial boundaries α/γ’ and γ’ε as a function of temperature.

In addition, Michalski and Wolowiec-Korecka [29,30] found that nitrogen potential can perform the function of controlling the nitriding process only in the nitriding atmospheres obtained from a one-component ammonia atmosphere, HN_3_, and those obtained from a two-component atmosphere (ammonia and dissociated ammonia NH_3_*/*NH_3diss_) at atmospheric pressure. They showed analytically that for processes carried out in nitriding atmospheres obtained from a two-component atmosphere with nitrogen and carried out at reduced pressure, there is no direct relationship between the value of the nitrogen potential and the degree of nitrogen dilution of the ingoing atmosphere. An original method was proposed for characterizing nitriding atmospheres, obtained from a two-component ingoing atmosphere with nitrogen at reduced pressure and atmospheric pressure, using the degree of dissociation of ammonia.

The article [31] discusses the problem of modifying the activity of nitriding media by diluting ammonia with nitrogen and the accompanying change in the degree of ammonia dissociation. The effects of these parameters on the growth kinetics of the nitrided layer and the phase composition of the near-surface iron nitride layer were investigated, finding that the nitriding temperature and the degree of nitrogen dilution of ammonia have a statistically significant effect on the growth kinetics of the nitrided layer. They also found that the degree of dissociation of ammonia and the degree of nitrogen dilution of ammonia significantly affect the value of nitrogen potential and thus the nitrogen concentration on the nitrided surface.

## 3. Nitrogen Potential as a Function of Hydrogen Content in the Nitriding Atmosphere

The concept of nitrogen potential was introduced by B.J. Lightfoot and D.H. Jack in 1975 [32] as a physicochemical parameter determining the gas nitriding process, expressed by the following relationship:

(1) $$N_{p}=\frac{p_{NH_{3}}}{p_{H_{2}}^{3/2}}$$

where $p_{NH_{3}}$ and $p_{H_{2}}$ are partial pressures of ammonia and hydrogen in the nitriding (exhaust) atmosphere, respectively.

Nitrogen potential characterizes the equilibrium state of nitrogen concentration between the nitriding atmosphere and the steel surface. At the same time, it is not a parameter that determines the kinetics of the process. However, knowledge of its value greatly facilitates the selection of optimal variants of nitriding atmospheres in terms of their type and phase composition of the near-surface layer of iron nitrides.

### 3.1. Single-Component NH_3_ Ingoing Atmosphere

To calculate the value of the nitrogen potential according to the relationship (1), one needs to know the partial pressures of ammonia or hydrogen. If it is assumed that the components of the nitriding atmosphere are a mixture of perfect gases, then according to Dalton’s law, the partial pressures of the components of the nitriding atmosphere correspond to the volume contents (shares) in the atmosphere. Thus, the formula for calculating the nitrogen potential has the following form:

(2) $$N_{p}=\frac{n}{{\left(Q\right)}^{3/2}}$$

where *n* is the volumetric content of ammonia in the (outgoing) nitriding atmosphere and *Q* is the volumetric content of hydrogen in the (outgoing) nitriding atmosphere.

**Figure 1:**
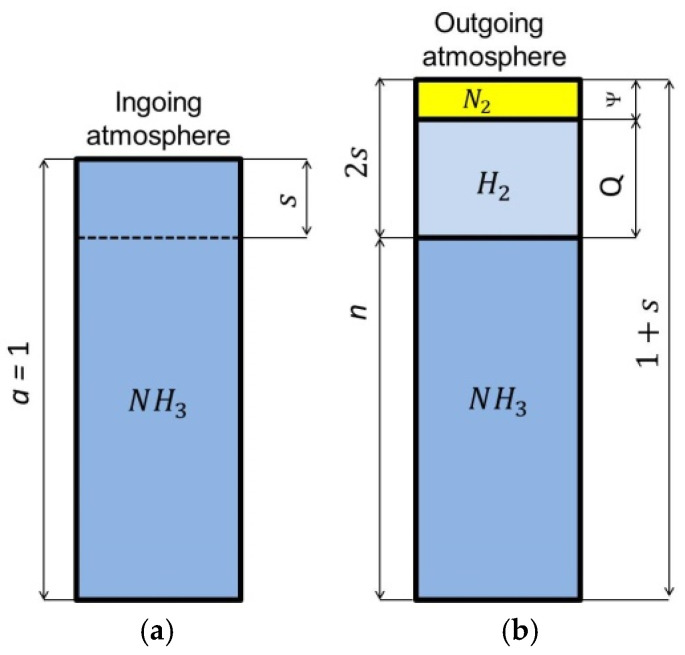
Schematic of single-component ingoing atmosphere (**a**) and outgoing atmosphere (**b**).

Figure 1 shows schematically the composition of the single-component ingoing atmosphere and the outgoing atmosphere, after dissociation of the ammonia parts of the ingoing atmosphere.

To calculate the nitrogen potential value, the volumetric content of ammonia or hydrogen in the outgoing atmosphere must be known. The required volumetric shares of ammonia and hydrogen can be calculated from the formulas:

- For ammonia

(3) $$n=\frac{a-s}{1+s}$$

- For hydrogen

(4) $$Q=\frac{1.5\cdot s}{1+s}$$

where *a* is the content of ammonia in the ingoing atmosphere (for a single-component ammonia atmosphere *a* = 1), *n* is the content of ammonia in the outgoing atmosphere that has not dissociated, and *s* is the content of ammonia in the ingoing atmosphere that will dissociate during the nitriding process.

When measuring the hydrogen content (*Q*) in the outgoing atmosphere, the value of s is calculated from Equation (5) after transforming Equation (4):

(5) $$s=\frac{Q}{1.5-Q}$$

The ammonia content of the outgoing atmosphere *n* is calculated from Equation (6) after substituting expression (5) into Equation (3):

(6) $$n=1-\frac{4}{3}Q$$

By substituting expression (5) into Equation (2), we obtain the formula for calculating the nitrogen potential for a single-component ammonia atmosphere:

(7) $$N_{p}=\frac{1-\frac{4}{3}Q}{{\left(Q\right)}^{3/2}}$$

At a constant temperature and atmospheric flow rate in the furnace retort, a local equilibrium between ammonia and hydrogen is established, which is described by the nitrogen potential. If the ingoing atmosphere is a single-component ammonia atmosphere, one potential value corresponds to only one hydrogen content in the nitriding atmosphere. For ingoing ammonia atmospheres, E. Lehrer [33] determined for iron the areas of solution persistence of αFe(N) and γ’-Fe_4_N, and ε-Fe_3_N depending on the temperature and ammonia content of the nitriding atmosphere, and then determined the boundaries of the areas of occurrence of each phase. The boundary potential relationships $N_{p}^{\alpha /\gamma \prime }(T)$ and $N_{p}^{\gamma \prime /\varepsilon }(T)$ describe the boundaries separating the α/γ’ and γ’/ε persistence areas, respectively:

(8) $$N_{p}^{\alpha /\gamma \prime }(T)=318.2766\cdot exp\left(\frac{40536}{T}-12.88\right)\;\left[{atm.}^{-0.5}\right]$$

(9) $$N_{p}^{\gamma \prime /\varepsilon }(T)=318.2766\cdot exp\left[{\left(\frac{60536}{T}-56.85\right)}^{0.5}-9.63\right]$$

**Figure 2:**
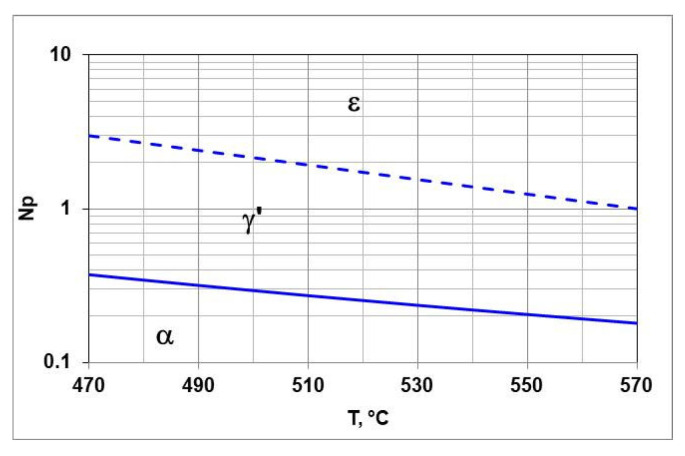
T-N_p_, balance, boundary potential $N_{p}^{\alpha /\gamma \prime }$ solid line, $N_{p}^{\gamma^{\prime }/\varepsilon }$ dashed line.

Figure 2 shows the balance T-Np. It marks the areas of persistence of αFe(N) and γ’-Fe_4_N and ε-Fe_3_N and the boundary potentials $N_{p}^{\alpha /\gamma \prime }$ and $N_{p}^{\gamma \prime /\varepsilon }$. The Lehrer chart can be used to design the steel nitriding processes.

To calculate the hydrogen content required to achieve a given nitrogen potential, one must solve Equation (7) for *Q*. The solution of the equation is the following function:

(10) $$Q\left(N_{p}\right)\approx 0.75\cdot \left\{u\cdot {\left(N_{p}-v\right)}^{w}\right\}$$

where *u =* 987026, *v* = −0.98799, *w* = −59265.

If we insert the boundary potentials $N_{p}^{\alpha /\gamma \prime }$ and $N_{p}^{\gamma \prime /\varepsilon }$ into Equation (10) in place of *N_p_*, we obtain formulas for calculating the hydrogen limits $Q^{\alpha /\gamma \prime }$ and $Q^{\gamma^{\prime }/\varepsilon }$ as a function of temperature, which corresponds to the nitrogen limits $N_{p}^{\alpha /\gamma \prime }\left(T\right)$ and $N_{p}^{\gamma \prime /\varepsilon }\left(t\right)$ of the Lehrer system:

(11) $$Q^{\alpha /\gamma^{\prime }}(T)\approx =0.75\cdot \left\{987026\cdot {\left(N_{p}^{\alpha /\gamma \prime }+0.98799\right)}^{-59265}\right\}$$

(12) $$Q^{\gamma^{\prime }/\varepsilon }(T)\approx 0.75\cdot \left\{987026\cdot {\left(N_{p}^{\gamma \prime /\varepsilon }+0.98799\right)}^{-59265}\right\}$$

**Figure 3:**
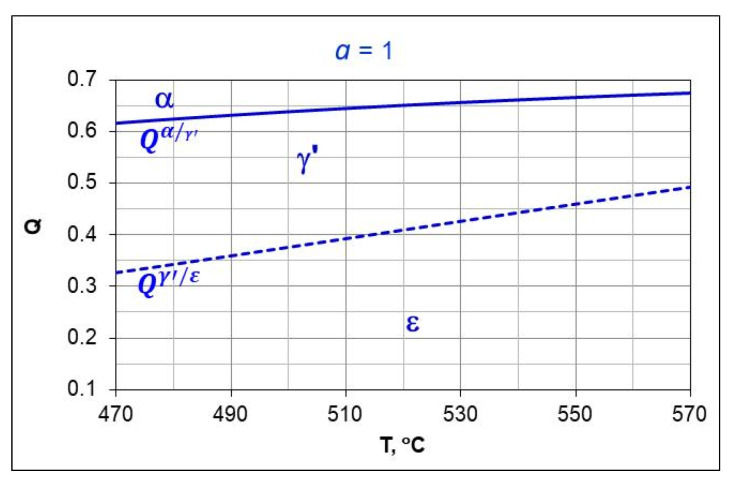
T-Q balance, boundary potential $N_{p}^{\alpha /\gamma \prime }$ solid line, $N_{p}^{\gamma^{\prime }/\varepsilon }$ dashed line.

Figure 3 shows the T-Q balance, marks the areas of persistence αFe(N) and γ’-Fe_4_N i ε-Fe_3_N, and the boundary hydrogen contents of the nitriding atmosphere $Q^{\alpha /\gamma \prime }$ $Q^{\gamma \prime /\varepsilon }$. The boundary hydrogen contents correspond to the boundary potentials $N_{p}^{\alpha /\gamma \prime }$, $N_{p}^{\gamma \prime /\varepsilon }$, respectively.

### 3.2. Two-Component Ingoing Atmosphere aNH_3_/N_2_

**Figure 4:**
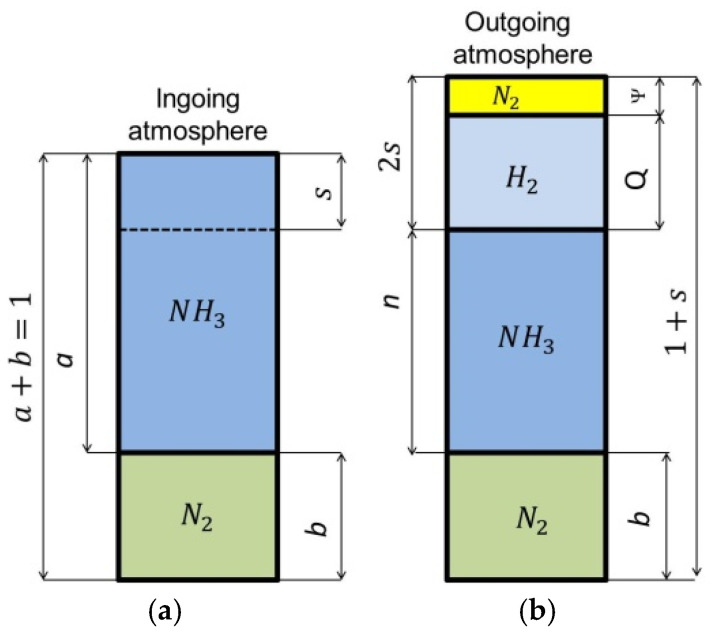
Schematic of two-component ingoing atmosphere (*a*NH_3_*/b*N_2_) (**a**) and outgoing atmosphere (**b**).

Figure 4 shows schematically the composition of the two-component ingoing atmosphere NH_3_/N_2_ and the outgoing atmosphere after dissociation of the ammonia parts of the ingoing atmosphere.

For a two-component ingoing atmosphere of *a*NH_3_*-b*N_2_, the dependence of the nitrogen potential value on the hydrogen content (*Q*) of the outgoing atmosphere and the ammonia content *(a*) is described by the function [29]:

(13) $$N_{p}\left(Q,a\right)=\frac{a\cdot \left(1-\frac{2}{3}Q\right)-\frac{2}{3}Q}{{\left(Q\right)}^{\frac{3}{2}}}$$

where *a* is the volumetric share of ammonia in the ingoing atmosphere (0<a≤1) and *b* is the volumetric share of nitrogen in the ingoing atmosphere (*a* + *b* = 1).

**Figure 5:**
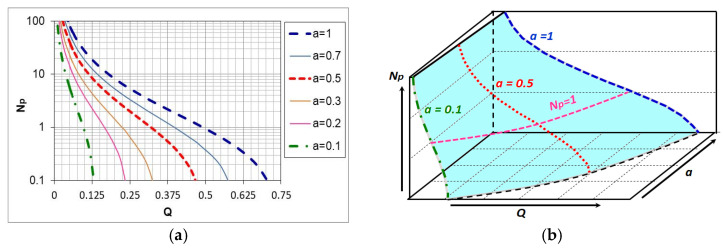
The course of changes in the nitrogen potential value of the nitriding atmosphere depending on the hydrogen content of the outgoing atmosphere for selected ammonia contents in the ingoing atmosphere *a*NH_3_*/b*N_2_ (**a**), surface *N_p_*(Q,b) in the system *(Q-a-Np*) (**b**) [5].

Equation (13) is a function of two variables, the graphical form of which is a surface in the coordinate system: hydrogen content in the outgoing atmosphere, ammonia content in the ingoing atmosphere *a*NH_3_*/b*N_2_, nitrogen potential (*Q-a-N_p_*) (Figure 5).

As can be seen in Figure 5, unlike the single-component ammonia ingoing atmosphere, different ammonia contents in the two-component ingoing atmosphere can correspond to a single potential value (*N_p_* = 1) and different hydrogen contents in the outgoing atmosphere (line *N_p_* = 1, Figure 5a). At the same time, one value of hydrogen (*Q* = 0.25) can correspond to different values of the nitrogen potential (line *Q* = 0.25, Figure 5a). By solving Equation (11) against *Q*, we obtain the dependence of the hydrogen content of the nitriding atmosphere on the nitrogen potential and the ammonia content (*a*) in the ingoing atmosphere *a*NH_3_*/b*N_2_. The general form of the function is the same as for the single-component ammonia ingoing atmosphere, with the difference that the values of the coefficients *u*, *v*, and *w* of the equation are a function of the ammonia content (*a*) in the ingoing atmosphere:

(14) $$Q\left(N_{p},a\right)\approx 0.75\cdot u\left(a\right)\cdot {\left(N_{p}+v\left(a\right)\right)}^{w\left(a\right)}$$

where: *u*(*a*), *v*(*a*), *w*(*a*)—experimental coefficients, *a*—ammonia content in the two-component ingoing atmosphere *a*NH_3_*/b*N_2_ (*a + b* = 1).

By substituting the relationships (8) and (9) into Equation (14), we obtain functions for calculating the temperature-dependent hydrogen content limits $Q^{\alpha /\gamma \prime }$ γ’ and $Q^{\gamma^{\prime }/\varepsilon }$, which correspond to the nitrogen limit potentials $N_{p}^{\alpha /\gamma \prime }\left(T\right)$ and $N_{p}^{\gamma \prime /\varepsilon }\left(t\right)$ of the Lehrer system.

(15) $$Q^{\alpha /\gamma \prime }\left(T,a=0.1\right)\approx 0.75\cdot \left\{0.207555\cdot {\left(N_{p}^{\alpha /\gamma^{\prime }}\left(T\right)+1.26086\right)}^{-0.59058}\right\}$$

(16) $$Q^{\gamma^{\prime }/\varepsilon }\left(T,a=0.1\right)\approx 0.75\cdot \left\{0.207555\cdot {\left(N_{p}^{\gamma \prime /\varepsilon }(T)+1.26086\right)}^{-0.59058}\right\}$$

(17) $$Q^{\gamma^{\prime }/\varepsilon }\left(T,a=0.5\right)\approx 0.75\cdot \left\{0.625405\cdot {\left(N_{p}^{\alpha /\gamma^{\prime }}\left(T\right)+0.9065\right)}^{-0.59244}\right\}$$

(18) $$Q^{\gamma^{\prime }/\varepsilon }\left(T,a=0.5\right)\approx 0.75\cdot \left\{0.625405\cdot {\left(N_{p}^{\gamma \prime /\varepsilon }(T)+0.9065\right)}^{-0.59244}\right\}$$

**Figure 6:**
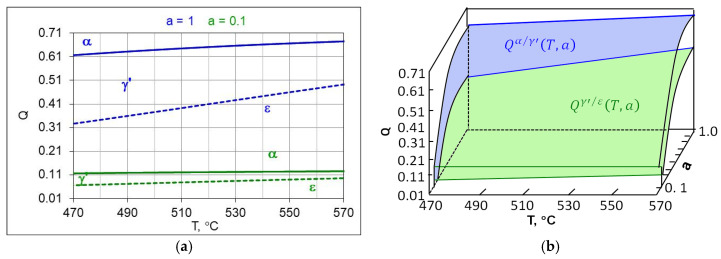
The modified Lehrer system (**a**) in the T-Q setup, for the ingoing atmosphere of 50%NH_3_/50%N_2_ (*a* = 0.5) (**a**) and an ingoing atmosphere of 10%NH_3_/N_2_ (*a* = 0.1) (**b**), $Q^{{\alpha /\gamma }^{\prime }}$ continuous line, $Q^{{\alpha /\gamma }^{\prime }}$ dashed line, (**b**) the boundary hydrogen content of the nitriding atmosphere, forming surface $Q^{{\alpha /\gamma }^{\prime }}(T,a)$ blue and ($Q^{\gamma \prime /\varepsilon }(T,a)$ green color.

Figure 6 shows the T-Q balance for an ingoing atmosphere of 50%NH_3_/50%N_2_ (*a* = 0.5) (Figure 6a) and an ingoing atmosphere of 10%NH_3_/N_2_ (*a* = 0.1).

Each composition of the ingoing atmosphere *a*NH_3_*/b*N_2_ corresponds to a separate T-*Q* balance. As the dilution of the ingoing atmosphere with nitrogen increases, the boundaries of $Q^{\gamma^{\prime }/\varepsilon }$ and $Q^{\alpha /\gamma \prime }$ shift toward lower hydrogen contents in the nitriding atmosphere, the α persistence area also grows while the γ’ and ε persistence areas are reduced.

### 3.3. Nitrogen Availability of the Nitrogenizing Atmosphere

The nitriding process is carried out under conditions of forced flow of nitriding atmosphere. From a practical point of view, it is important to identify the factors determining the supply of nitrogen obtained from the ammonia dissociation reaction. The amount of nitrogen involved in the formation of the nitrided layer depends on temperature, flow rate, and composition of the nitriding atmosphere. The kinetics and phase composition of the nitrided layer that forms are determined by the relationship between the nitrogen flux from the atmosphere to the steel surface and the nitrogen flux that diffuses deep into the steel. The first flux describes the law of mass transport in the nitriding atmosphere, while the second one describes the laws of diffusion in steel. During the nitriding process, only the nitrogen flow from the nitriding atmosphere to the nitrided steel surface can be regulated in a controlled manner. There are two basic chemical reactions in the nitriding process [34]:

(19) $${NH}_{3(g)}\overset{Fe}{\to }\frac{1}{2}N_{2(g)}+\frac{3}{2}H_{2(g)}$$

(20) $${NH}_{3(g)}\overset{Fe}{\to }{\left[N\right]}_{\alpha }+\frac{3}{2}H_{2(g)}$$

where [*N*]*_α_* is nitrogen dissolved in the α-phase.

At the process temperature in thermodynamic equilibrium, the degree of thermal dissociation of ammonia is very close to 1. However, slow thermal dissociation kinetics (Equation (19)) with a sufficiently high gas flow rate result in a steady state for the furnace atmosphere, which is characterized by the ammonia dissociation of much less than 1. This allows nitriding to proceed according to reaction (20) [35].

As a result of the dissociation reaction (reaction (19), 0.5 mole of nitrogen and 1.5 mole of hydrogen are formed from one mole of ammonia. Knowing the volume of dissociating ammonia, the mass of a mole of nitrogen (28.016 g), and its volume (22.414 dm^3^), one can calculate the mass of nitrogen obtained from the ammonia dissociation reaction:

(21) $$m_{N_{2}}=0.5V_{NH_{3}}^{*}\cdot \frac{28.016}{22.414}≅0.625\cdot V_{NH_{3}}^{*}$$

where $V_{NH_{3}}^{*}$ is the volume of ammonia that dissociates during the nitriding process.

The volume fraction of ammonia *s* in the inlet atmosphere that undergoes dissociation is expressed by the relation:

(22) $$s=\frac{V_{NH_{3}}^{*}}{V_{in}}$$

where *V_in_* is *the* volume of the ingoing atmosphere.

We will calculate the volume of ammonia that dissociates, by transforming Equation (22):

(23) $$V_{NH_{3}}^{*}=s\cdot V_{in}$$

The mass of nitrogen obtained from the ammonia dissociation reaction can be calculated by substituting Equation (23) into Equation (21):

(24) $$m_{N_{2}}≅0.625\cdot s\cdot V_{in}$$

By substituting in place of the volume of the ingoing atmosphere ($V_{in})$ flow rate ($F_{in})\;$ in dm^3^/min, we will calculate the mass of nitrogen in g/min:

(25) $$m_{N_{2}}\left(t\right)≅0.625\cdot s\cdot F_{in}$$

By substituting the relationship (5) into Equation (25), we obtain a formula that allows us to calculate the availability of nitrogen as a function of time and the hydrogen content of the outgoing atmosphere:

(26) $$m_{N_{2}}\left(t\right)≅0.625\cdot \frac{Q(t)}{1.5-Q(t)}\cdot F_{in}$$

The availability of nitrogen, at a constant flow rate of the ingoing atmosphere, is a function of the hydrogen content of the nitriding/outgoing atmosphere. As the nitrogen content of the ingoing atmosphere changes, the hydrogen content of the nitriding atmosphere also changes and, consequently, so does the availability of nitrogen. However, this change is not accompanied by a change in the value of the nitrogen potential (Figure 5) [4].

## 4. Materials and Methods

### 4.1. Characteristics of Steel Used in the Tests

Nitriding processes were carried out on samples made of AISI 52100 steel in the shape of a ball with a diameter of 3 mm. Identical balls are used in rolling bearings. The chemical composition of the steel is shown in Table 1. In addition to carbon and the usual admixtures, the AISI 52100 steel contains 1.5% by weight of chromium. Chromium is an element with a high affinity for nitrogen; the use of steel balls allowed us to study how the presence of chromium in steel would affect the kinetics of the increase in the thickness of the iron nitride layer during nitriding and the thickness of the solution zone. The advantage of the bearing balls used, in addition to the very low unit price, is the repeatability of the unit weight, which is very important in thermographic testing. The reproducible actual surface of the balls is also not insignificant, as they are polished.

### 4.2. Characteristics of the Nitriding Device

**Figure 7:**
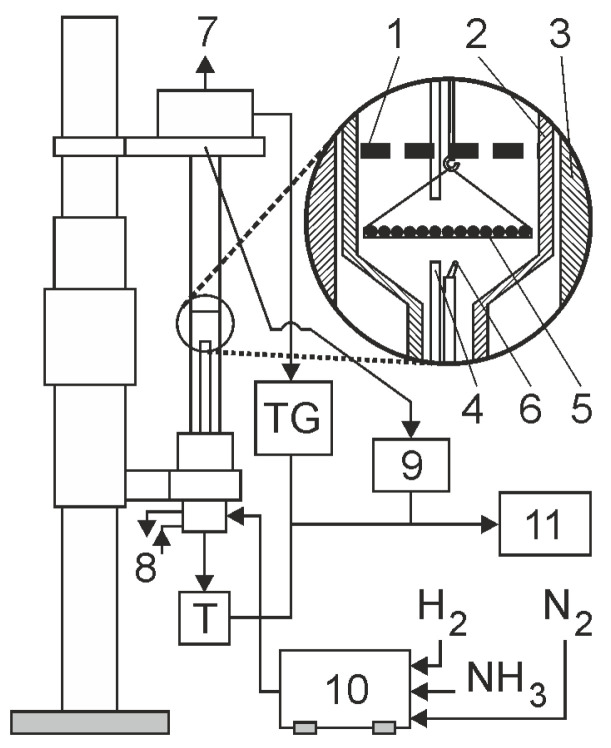
General diagram of the functioning of a thermobalance, 1—shutter; 2—reactor wall; 3—reactor furnace; 4—gas phase sampling point; 5—sample holder with single layer of grains; 6—thermocopule; 7—vent; 8—cooling water (closed circuit); 9—hydrogen analyzer; 10—gas mixer; 11—process control computer.

Nitriding processes were carried out in a chemical reactor (Figure 7). The reaction chamber is made of a quartz tube with an inner diameter of 28 mm. During nitriding, the flow rate of the nitriding mixture was controlled, and the process temperature, hydrogen content of the outgoing atmosphere, and the change in the mass of the nitrided samples were recorded. Twenty samples of the total mass of 2204.21 ± 0.35 mg were nitrided at one time. The samples were arranged on a dish, made of platinum mesh, in a single layer. Platinum ensured that only the weight gain of the steel samples was recorded during the nitriding process, while the mesh used in the basket did not inhibit the flow of the nitriding atmosphere. The accuracy of weight change registration was ± 0.5 mg. In all the experiments, the flow rate of the nitriding mixture was 0.2 dm^3^/min.

### 4.3. Characteristics of Nitriding Process Parameters

#### 4.3.1. Analysis of the Error in Assessing the Value of the Nitrogen Potential

In two-component process atmospheres *a*NH_3_/*b*N_2_, where *a* and *b* are the volume shares of ammonia and nitrogen, respectively. The accuracy of assessing the nitrogen potential value depends on the accuracy of measuring the hydrogen content *(Q*) in the outgoing atmosphere and the accuracy of assessing the degree of nitrogen dilution of ammonia (*a*). The atmospheres used in the tests were NH_3_ (*a* = 1), 0.5NH_3_/0.5N_2_ (*a* = 0.5), 0.1NH_3_/0.9N_2_ (*a* = 0.1). Taking this into account, the uncertainties of the nitrogen potential determination were calculated for all ingoing atmosphere compositions, in the *a*NH_3_*/b*N_2_ atmosphere, as *a* function of hydrogen content in the outgoing atmosphere.

When the *a*NH_3_/*b*N_2_ atmosphere was used, the nitrogen potential value was calculated using Equation (5). It was assumed that the uncertainty of the calculated values of nitrogen potential *(N_p_*) is the sum of the square roots of the uncertainties in determining the independent variables *(a* and *Q*):

(27) $$U_{N_{p}}=\pm {\left[{\left(u_{a}\frac{\partial \left(N_{p}\right)}{\partial a}\right)}^{2}+{\left(u_{Q}\frac{\partial \left(N_{p}\right)}{\partial Q}\right)}^{2}\right]}^{0.5}$$

where *u_a_* = uncertainty of determination of *a* (±), *u_Q_* = uncertainty of determination of *Q* (±), and *U_Np_* = total uncertainty of determination of the value of nitrogen potential (±).

By substituting Equation (13) into expression (27) and differentiating, we obtain the relationship for calculating the error of estimating the value of the nitrogen potential as a function of the variables *a* and *Q*:

(28) $$U_{N_{p}}=\pm {\left\{{\left[u_{a}\left(Q^{-1.5}-\frac{2}{3}Q^{-0.5}\right)\right]}^{2}+{\left[u_{a}\left({\frac{a+1}{3}Q}^{-1.5}-\frac{3}{2}{a\cdot Q}^{-2.5}\right)\right]}^{2}\right\}}^{0.5}$$

**Figure 8:**
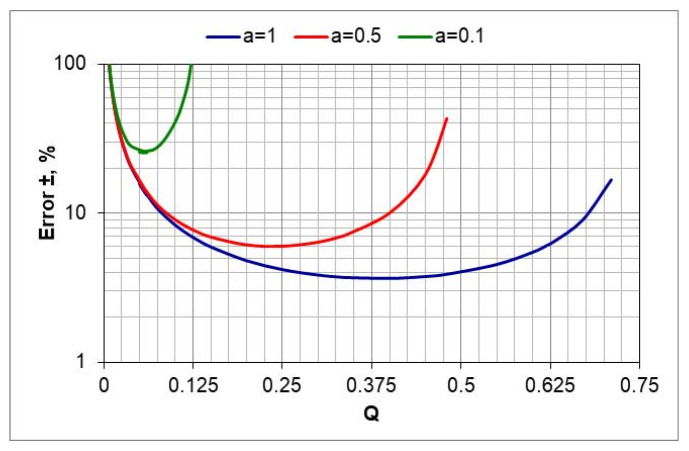
The dependence of the estimation error of the nitrogen potential value as a function of the variables *a* and *Q* (the uncertainties of the determination of *a* and *Q* are, respectively, *u_a_* = ±0.01 and *u_Q_* = 0.005.

The calculations show that in the case of a nitrogen-diluted ingoing atmosphere, the largest error in estimating the value of nitrogen potential can occur when using the 10%NH_3_/90%N_2_ atmosphere—its value increases as the temperature decreases from 30% to 40% at 570 and 510 °C, respectively (Figure 8, *a* = 0.1).

#### 4.3.2. Nitriding Process Parameters

Process temperatures correspond to those used in industrial processes for both alloyed and unalloyed steels. In practice, NH_2_/N_2_ atmospheres are also used, with up to 50–60% of nitrogen content, where the rest is ammonia. For comparison, the tests used the 10%NH_3_/90%N_2_ atmosphere (Table 2).

### 4.4. Metallographic Tests on the Nitrided Samples

**Figure 9:**
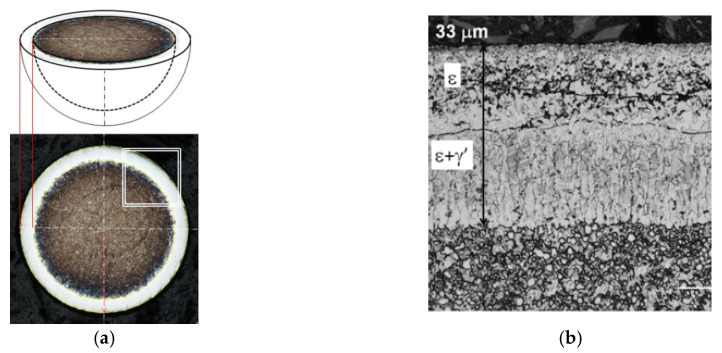
Diagram for measuring the thickness of the iron nitride (WL) layer of a sphere. (**a**) The gray (above) and white (below) surfaces represent the iron nitride layer; g_mp_ represents the actual thickness of the iron nitride (WL) layer, and D_K_ represents the diameter of the sphere, the appearance of the sphere before and after encapsulation and grinding (**b**).

Metallographic tests were performed on the specimens hot-encapsulated in thermosetting resin. The encapsulated balls were ground to their diameter, which made it possible to observe an iron nitride layer of actual thickness. Figure 9a shows the method of measuring the thickness of the iron nitride layer, and Figure 9b shows the appearance of the sphere before and after encapsulation and grinding.

### 4.5. Tests of the Phase Composition of Layers and Nitrogen Concentration of Iron Nitrides by XRD and EDS

Iron nitride layers on the samples were determined by X-ray diffraction using a Seifert 3003TT X-ray diffractometer (Freiberger Präzisionsmechanik GmbH, Freiberg, Germany), with CoKα radiation and a symmetrical measurement geometry. The range of recorded diffraction angles of 40–58° included the main characteristic lines of γ’ and ε iron nitrides and iron according to the standards from the PDF4 diffractometer database.

The morphology and nitrogen concentration on the surface of the nitrided layer were tested using a Hitachi S-3400N scanning electron microscope (Tokyo, Japan). The accelerating voltage during the tests was 15 keV. The penetration depth of the beam was about 1 μm, and the detector area was 30 mm^2^. The chemical composition was studied using a scanning electron microscope equipped with an EDS X-ray analyzer from AMETEK GmbH (Berwyn, PA, USA). Uncertainty in measuring nitrogen concentration ± 0.5 wt.%.

## 5. Discussion of Research Results

### 5.1. Gravimetric Tests

**Figure 10:**
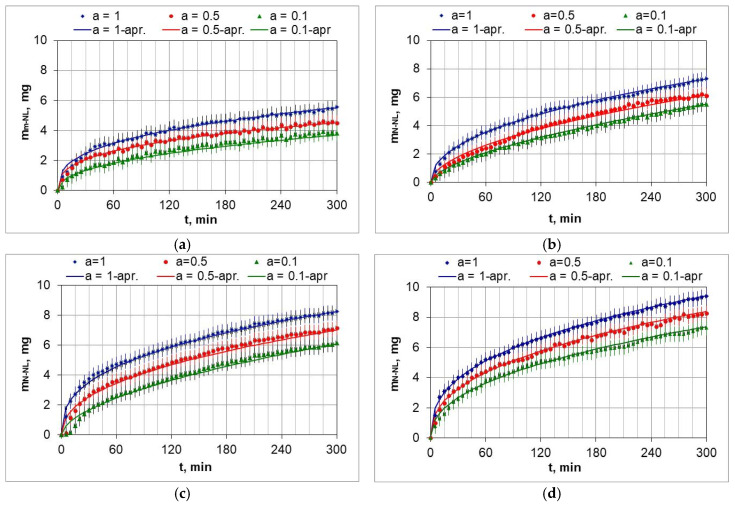
Mass change in the m_NL_ samples as a function of time of nitriding process at 510 °C (**a**), 530 °C (**b**), 550 °C (**c**), 570 °C (**d**), for ingoing atmosphere—NH_3_—*a* = 1, 0.5NH_3_/0.5*b*N_2_—*a* = 0.5, 0.1NH_3_/0.9*b*N_2_—*a* = 0.1 (*a* + *b* = 1).

Figure 10 shows the weight change in the AISI 52100 steel samples during nitriding in the temperature range 510–570 °C in nitriding atmospheres with different ammonia contents in the ingoing atmosphere. The lowest weight gains of the samples occurred at 510 °C and using the ingoing atmosphere of 10%NH_3_/90%N_2_ (Figure 10a, *a* = 0.1), with the highest at 570 °C when using the ammonia atmosphere (Figure 10d, *a* = 1).

The change in mass of the samples as a function of the time of the nitriding process can be approximated by a power function:

(29) $$m_{NL}(t)=k_{NL}\cdot t^{n}$$

The mass change function of the nitrided samples deviates from the parabolic course typical of diffusion-controlled processes. The function approximations also include the heating time of the samples from 470 °C to the process temperature. This may be one of the reasons for the deviations. The coefficients of the approximation functions of the mass change in the nitrided layer increase with increasing dilution of the ingoing atmosphere with nitrogen. Table 3 lists the coefficients of approximation functions of the mass change $m_{NL}(t),$ as a function of time according to the parameters given in Table 2.

### 5.2. Degree of Utilization of Process Atmosphere

**Figure 11:**
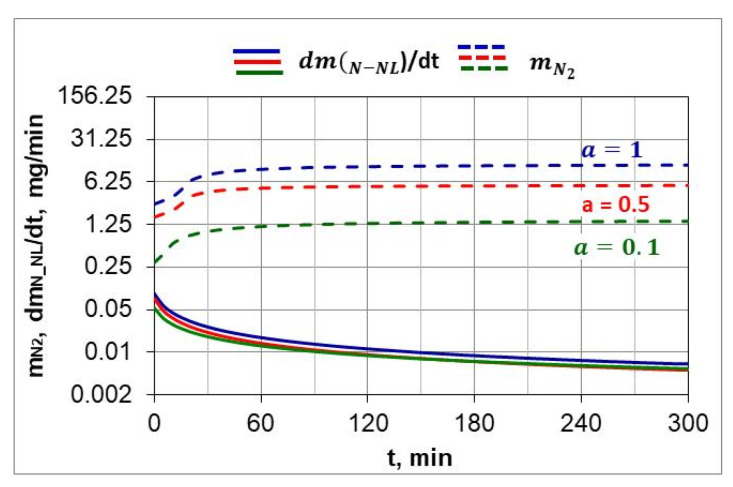
The change in the availability of nitrogen ($m_{N_{2}}$) during the nitriding process and the change in the absorption capacity of the ($d(m_{NL}(t)/d_{t})$ nitrided steel during the nitriding process for three compositions of the ingoing atmosphere, at 510 °C.

Following Simrnov and Kuleshov [28], a study of nitrogen utilization depending on nitriding temperature and ingoing atmosphere composition was conducted. Figure 11 shows an example of the change in nitrogen availability ($m_{N_{2}}$(*t*) during the nitriding process and the change in absorption capacity ${d(m}_{NL}(t)/dt\;$ of nitrided steel during the nitriding process at 510 °C for three ingoing atmosphere compositions. The change in nitrogen availability was calculated from relationship (26). As can be seen in Figure 11, nitrogen availability of the nitriding atmosphere increases at the initial stage of the process, i.e., when the charge is heated, and then its value is almost constant. In turn, the absorption capacity of nitrided steel decreases in accordance with the derivative function $m_{N-NL}(t)$. The availability of nitrogen far exceeds the absorption capacity of nitrided steel, the degree of utilization of nitrogen formed by the dissociation of ammonia decreases as the process proceeds and increases with the increase in the degree of nitrogen dilution of ammonia.

**Figure 12:**
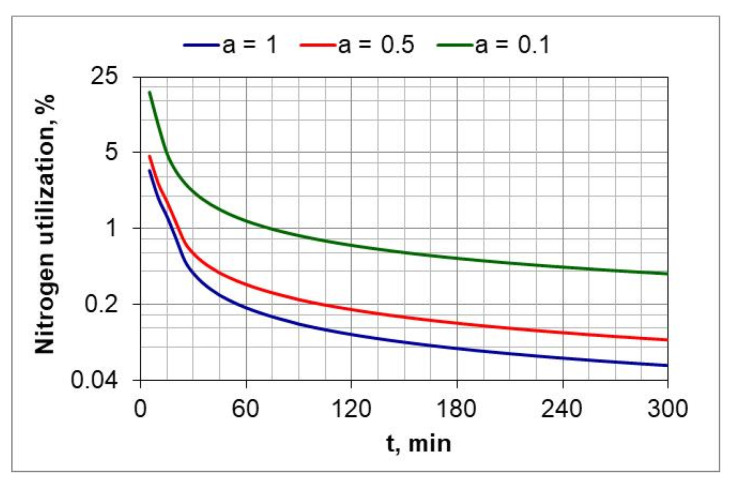
Change in nitrogen utilization during the nitriding process of AISI 52100 steel samples, for three compositions of ingoing atmosphere, at 510 °C.

Figure 12 shows the change in percentage utilization of nitrogen during nitriding. It is visible that nitrogen utilization does not exceed 1% after one hour of the process. In the ingoing atmosphere, NH_3_ (*a* = 1) and 50%NH_3_/50%N_2_ (*a* = 05), it decreases to below 0.2%.

**Figure 13:**
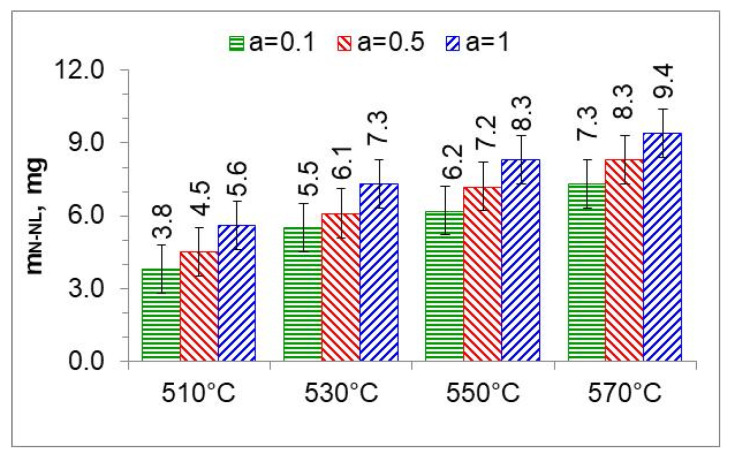
The mass of nitrogen in the nitrous layer ($m_{N-NL}$) formed at 510, 530, 550, and 570 °C for three ingoing atmosphere compositions.

**Figure 14:**
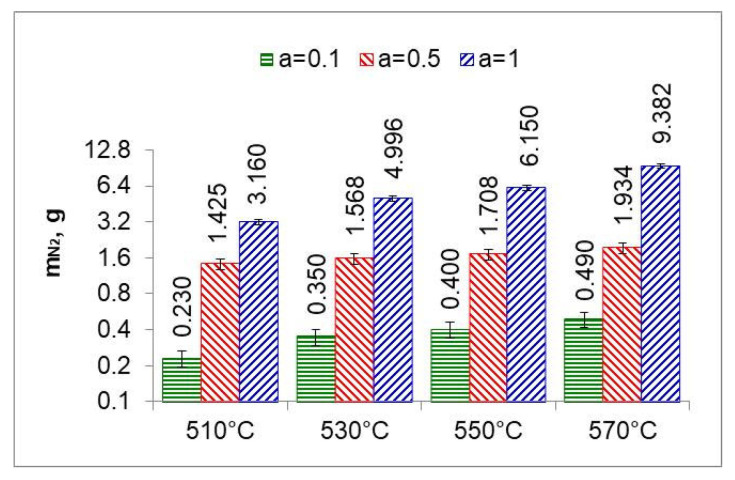
Nitrogen mass ($m_{N_{2}}$) produced in the nitriding process at temperatures of 510, 530, 550 and 570 °C for three ingoing atmosphere compositions.

By integrating the availability function (function (8)) $m_{N_{2}}$(t) in the limits (0–300 min), we obtain the mass of nitrogen formed during the nitriding process (Figure 13 and Figure 14).

**Figure 15:**
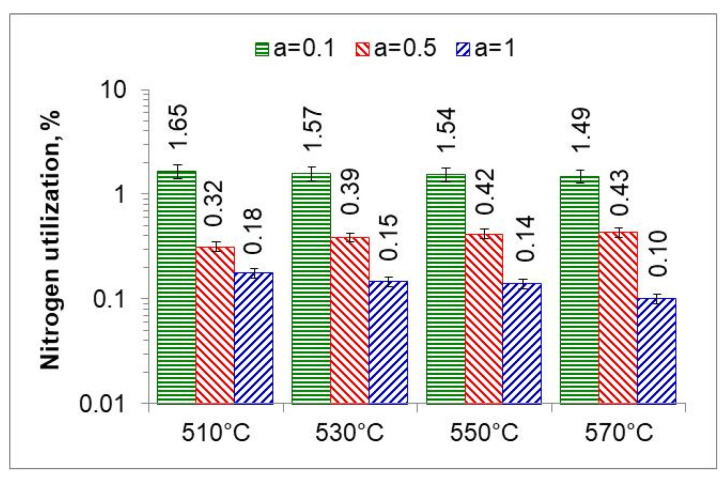
Nitrogen utilization in the nitriding process at temperatures of 510, 530, 550, and 570 °C for three ingoing atmosphere compositions.

The ratio of the mass of nitrogen in the nitrided layer $(m_{N-NL}$) to the mass of nitrogen ${(m}_{N_{2}})$ formed in the nitriding process is a measure of nitrogen utilization (U_N_) in the nitriding process (Figure 15). The nitrogen utilization rate was calculated from the relationship:

(30) $$U_{N}=\frac{m_{N-NL}}{\int_{0}^{300}m_{N_{2}}\left(t\right)dt}\cdot 100\%$$

where $m_{N-NL}$ is the mass of nitrogen in the nitrided layer and $m_{N_{2}}$ is the mass of nitrogen formed during the nitriding process due to dissociation.

The highest nitrogen utilization rate occurs at the greatest dilution of the ingoing atmosphere. The calculated nitrogen utilization rate is consistent with that reported by Smirnov [28]. The results of research in this area can be used for developing systems that adjust nitrogen availability in real time.

### 5.3. Microstructure Characterization

**Figure 16:**
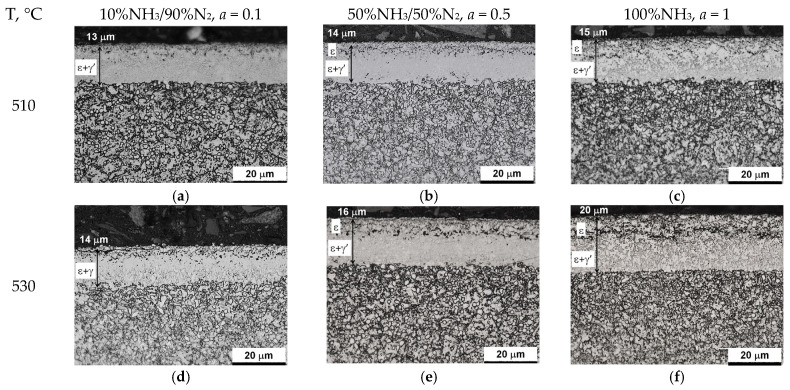

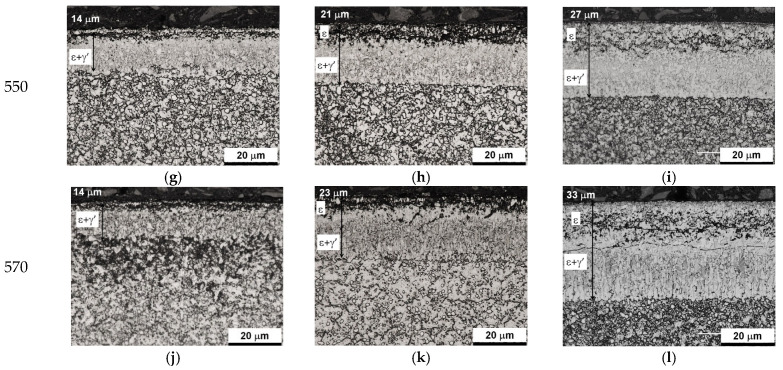
Microstructures of nitrided layers formed at 510, 530, 550, and 570 °C for three ingoing atmosphere compositions: 10%NH_3_/90%N_2_ (*a* = 0.1), 50%NH_3_/50%N_2_ (*a* = 0.5), 100%NH_3_ (*a* = 1).

Figure 16 shows the microstructures of nitrided AISI 52100 steel at 510, 530, 550, and 570 °C for three ingoing atmosphere compositions of 10%NH_3_/90%N_2_, 50%NH_3_/50%N_2_, and 100%NH_3_. The layers formed are of comparable thickness. In the near-surface zone of the iron nitride layers, there is a porous zone. The thickness of the porous zone increases as the thickness of the iron nitride layer increases. In turn, the thickness of the iron nitride layer increases with increasing temperature and ammonia content in the ingoing atmosphere. The exceptions are processes in the 10%NH_3_/90%N_2_ atmosphere (*a* = 0.1) where 141 m-thick iron nitride layers were obtained at all the temperatures (Figure 16).

As mentioned above, the growth of the nitrided layer on steel begins with the formation of the Feα(N) solution. Once the nitrogen concentration on the surface is established, corresponding to the persistence of the phase γ’, nucleation and growth of the γ’-Fe_4_N nitride on the surface of Feα(N) solution take place. If the value of the nitrogen potential does not exceed the value corresponding to the limit γ’/ε, coalescence of γ’ seeds and further growth of the monophasic γ’ layer [26] proceed. If, on the other hand, the potential takes values above the limit γ’/ε, phase ε will nucleate on the γ’ seeds. During nitriding at constant temperature, porosity development occurs due to the metastability of iron nitrides at a pressure of 1 atmosphere with respect to the equilibrium system of Fe and N_2_ [36].

**Figure 17:**
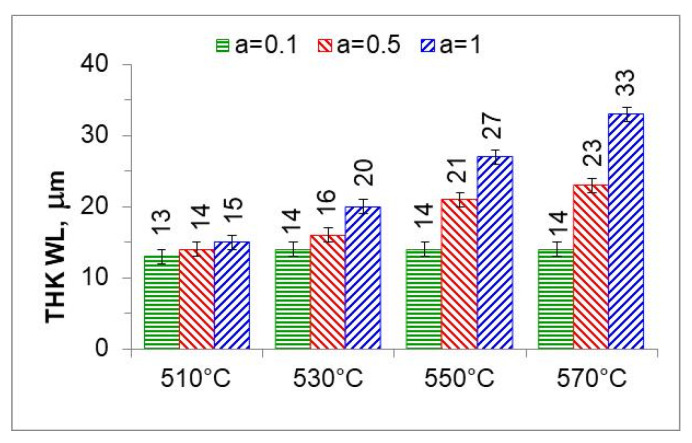
Thicknesses of iron nitride layers formed on AISI 52100 steel at 510, 530, 550, and 570 °C for three ingoing atmosphere compositions (10%NH_3_/90%N_2_, *a* = 0.1, 50%NH_3_/50%N_2_, *a* = 0.5, and 100%NH_3_, *a* = 1).

**Figure 18:**
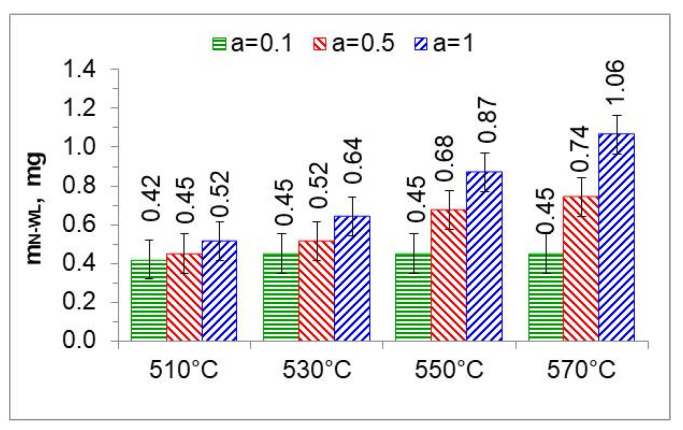
The mass of nitrogen in a layer of iron nitrides (*m_N-WL_*) at temperatures of 510, 530, 550, and 570 °C, in an atmosphere of 10%NH_3_/90%N_2_ (*a* = 0.1), 50%NH_3_/50%N_2_ (*a* = 0.5), and 100% NH_3_ (*a* = 1).

Figure 17 summarizes the thickness of iron nitride layers formed on AISI 52100 steel at 510, 530, 550, and 570 °C for three ingoing atmosphere compositions (10%NH_3_/90%N_2_ (*a* = 0.1) 50%NH_3_/50%N_2_ (*a* = 0.5) and 100%NH_3_ *a* = 1) and Figure 18 summarizes the mass of nitrogen in the iron nitride layers formed.

The growth of the nitrided layer on steel proceeds in multiple stages. The most important steps include adsorption of ammonia, chemical reactions of gradual dissociation of ammonia, absorption of the formed nitrogen, and its diffusion deep into the nitrided substrate. In the initial stage of film formation, the slowest stage is the chemical reaction of ammonia dissociation on the nitrided surface, known as the steel saturation stage. After the formation of the iron nitride layer, the slowest stage becomes the diffusion of nitrogen in steel. After the formation of the iron nitride layer, the nitrogen stream from the nitriding atmosphere is used to form the iron nitride layer and the solution zone. From this point on, the thickness of the iron nitride layer and the solution zone increase simultaneously.

In the nitriding atmosphere obtained from the ingoing atmosphere of 10%NH_3_/90%N_2_ (*a* = 0.1) at all temperatures, the mass of nitrogen absorbed in the iron nitride layer is the same, which indicates that with such nitrogen dilution of ammonia, the mass gain of nitrogen in the iron nitride layer is limited by the nitrogen availability of the nitriding atmosphere described by Equation (8). At 510 °C, nitrogen absorption can also be limited by the availability of nitrogen in the atmosphere of 50%NH_3_/50%N_2_ (*a* = 0.5). At temperatures of 550 and 570 °C and atmospheres of 100%NH_3_ (*a* = 1) and 50%NH_3_/50%N_2_ (*a* = 0.5), the increase in nitrogen mass in the iron nitride layer is limited by diffusion (Figure 18).

### 5.4. Change in the Mass of Nitrogen in the Iron Nitride Layer and in the Solution Zone Has Been Determined

**Figure 19:**
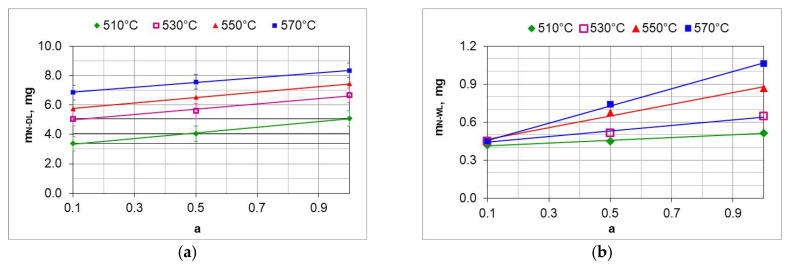
Change in the mass of nitrogen in the solution layer ($m_{N-SL})$ (**a**) and change in the mass of nitrogen in the iron nitride layer ($m_{N-WL})$ (**b**) as a function of ammonia content in the ingoing atmosphere.

The mass of nitrogen in the solution zone ($m_{N-SL})$ as a function of the ammonia content of the ingoing atmosphere varies linearly; the directional coefficients of the trend line for successive temperatures vary slightly (Figure 19a). It follows that the increase in nitrogen mass in the solution zone is limited by nitrogen diffusion.

The change in the mass of nitrogen in the iron nitride layer ($m_{N-WL})$ as a function of the ammonia content of the ingoing atmosphere runs differently from the increase in the mass of nitrogen in the solution zone (Figure 19b). At 510 °C, the mass of nitrogen in the iron nitride layer does not change with the increase in ammonia content in the ingoing atmosphere; the differences in the mass increase do not exceed 0.1 mg. This indicates that in a nitriding atmosphere obtained from an ingoing atmosphere with the composition of 10%NH_3_/90%N_2_ (*a* = 0.1), the change in the mass of nitrogen in iron nitride is limited by the availability of nitrogen. Above the temperature of 510 °C, the increase in nitrogen mass in the iron nitride layer may be limited by nitrogen diffusion.

### 5.5. Tests of Phase Composition and Nitrogen Concentration in Iron Nitrides

**Figure 20:**
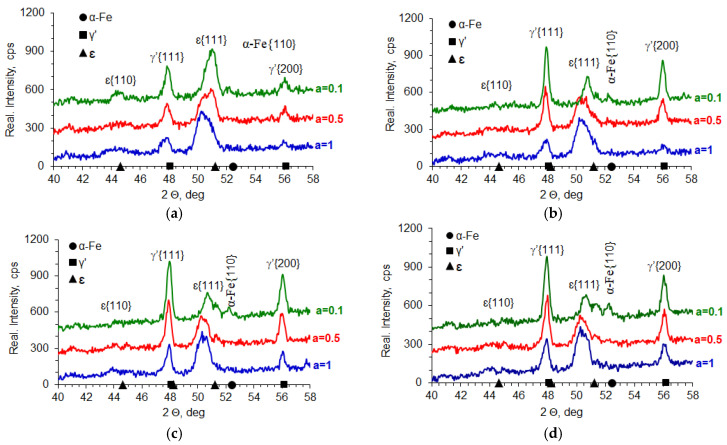
Results of X-ray analysis of samples nitrided at 510 °C (**a**), 530 °C (**b**), 550 °C (**c**), and 570 °C (**d**) using the ingoing atmosphere of 10%NH_3_/90%N_2_ (*a* = 0.1), 50%NH_3_/50%N_2_ (*a* = 0.5), and 100%NH_3_ (*a* = 1).

In the processes carried out at 510, 530, 550, and 570 °C using ingoing atmospheres of 10%NH_3_/90%N_2_ (*a* = 0.1), 50%NH_3_/50%N_2_ (*a* = 0.5), and 100%NH_3_ (*a* = 1), the nitrogen potential adopted values from the ε phase persistence region (Table 2). This is indicated by the X-ray tests of the surfaces of the nitrided samples. In diffractograms from the surfaces of all nitrided samples, there are lines characteristic of the ε and γ’ phases (Figure 20). Intensity of the peaks from the mentioned phases changes with temperature and the degree of nitrogen dilution of the ingoing atmosphere. As the temperature and the degree of dilution of the ingoing atmosphere increase, the amount of γ’ phase in the iron nitride layer increases. With an increase in nitriding temperature, at a constant intensity of the ingoing atmosphere, the value of the nitrogen potential decreases, and, according to the Lehrer diagram, the corresponding nitrogen surface concentration also decreases. Therefore, during cooling, according to the Fe-N balance, more phase γ’ is emitted. In the diffractograms, the intensities of the lines characteristic of the γ’{111} and γ’{200} planes increase (Figure 20). The increase in the amount of the γ’ phase as the dilution of the ingoing atmosphere with nitrogen increases is also due to the decrease in the surface concentration of nitrogen, but not as a result of the decrease in nitrogen potential, but as a result of the decrease in availability. Similar effects occur in vacuum nitriding, in which case the availability of nitrogen decreases as the partial pressure of ammonia in the nitriding atmosphere decreases as the pressure in the furnace chamber decreases.

**Figure 21:**
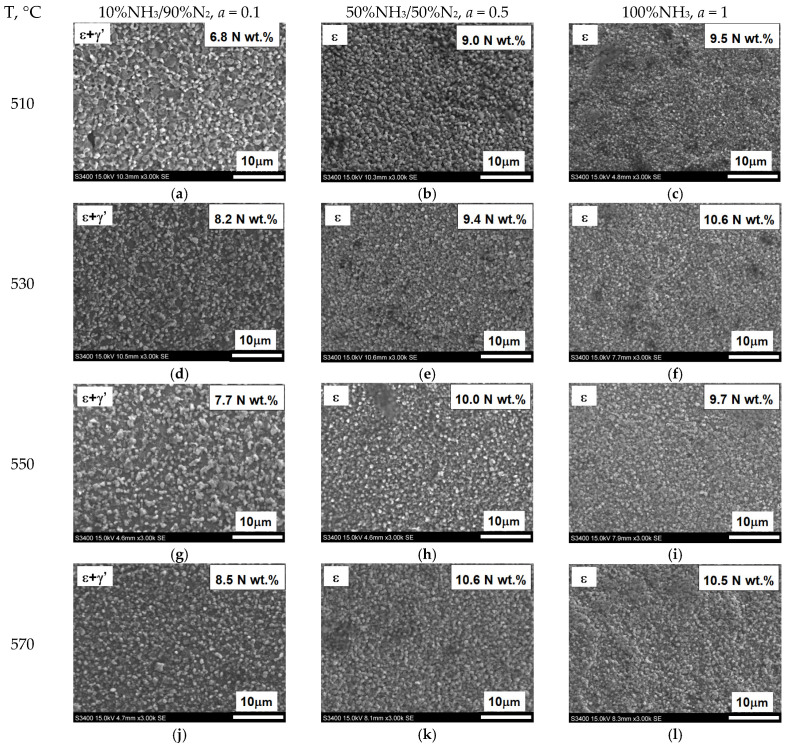
Surface appearance of nitrided AISI 52100 steel at 510, 530, 550, and 570 °C in an atmosphere of 100% NH_3_; (*a* = 1), 50%NH_3_/50%N_2_; (*a* = 0.5), 10%NH_3_/N_2_; (*a* = 0.1). The photographs show the nitrogen concentration in the near-surface zone of the iron nitride layer in weight percentage.

Figure 21 shows the surface appearance of AISI 52100 steel specimens nitrided at 510, 530, 550, and 570 °C in the nitriding atmospheres obtained from the ingoing atmosphere of 100%NH_3_ (*a* = 1), 50%NH_3_/50%N_2_ (*a* = 0.5), and 10%NH_3_/90%N_2_ (*a* = 0.1), the images show the average values of nitrogen concentration in the near-surface zone of the iron nitride layer. At steady-state temperature, in all processes, the potentials took on values from the phase persistence area ε. The concentration of nitrogen in the near-surface zone of iron nitrides formed by the 10%NH_3_/90%N_2_ atmosphere does not exceed 8.5% by weight (Figure 21a,d,g,j). The nitride layers formed under these conditions are a mixture of the ε and γ’ phases. In contrast, in the other samples (Figure 21b,c,e,f,h,i,k,l), the concentration in the near-surface zone of the nitrides exceeds 8.5% by weight; in this case, the iron nitride layers are two-zoned, where the zone near the surface is formed by the ε phase and below it—by the ε + γ zone.

### 5.6. Analysis of Process Parameters

**Figure 22:**
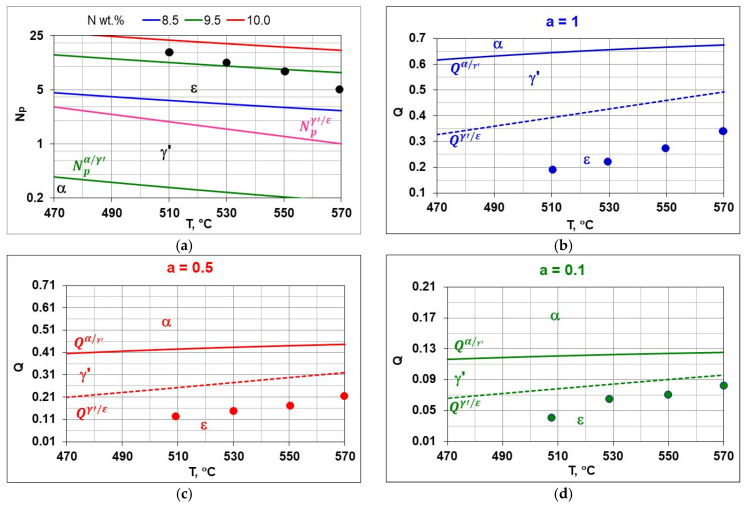
Lehrer diagram, with the points indicating the potential values for the temperatures of the processes performed (**a**), the boundary hydrogen contents of the nitriding atmosphere $Q^{\alpha /\gamma^{\prime }}$ and $Q^{\gamma^{\prime }/\varepsilon }$ for a single-component ingoing atmosphere of 100% NH_3_ (*a* = 1) (**b**), a two-component ingoing atmosphere with a nitrogen composition of 50%NH_3_/50%N_2_ (*a* = 0.5) (**c**), with a composition of 10%NH_3_/90%N_2_ (**d**), the boundary hydrogen contents of the $Q^{\alpha /\gamma^{\prime }}$ and $Q^{\gamma^{\prime }/\varepsilon }$ correspond to the boundary nitrogen potentials $N_{p}^{\alpha /\gamma \prime }$ and $N_{p}^{\gamma \prime /\varepsilon }$.

Figure 22d shows a Lehrer diagram in the T–N_p_–N system, presenting the equilibrium between the NH_3_+H_2_ atmosphere. In the temperature range of 470–570 °C. Ammonia in equilibrium conditions dissociates almost completely into hydrogen and nitrogen according to reaction (19). In order to obtain the assumed potential values, it is necessary to ensure a constant flow of atmosphere through the furnace chamber. The potential values under such conditions do not present thermodynamic equilibrium but a stationary state. Hence, there are differences in the values of nitrogen concentrations measured on samples after nitriding processes from the predicted nitrogen concentrations based on the recorded values of nitrogen potentials. The differences may also be due to insufficient accuracy in determining the hydrogen content of the nitriding atmosphere, the ammonia content of the NH_3_/N_2_ ingoing atmosphere, and the measurement of nitrogen concentration in the near-surface zone of the iron nitride layer. The modified graph shows the potential values calculated according to Equation (13); as can be seen from this graph, the values of nitrogen potentials took values in the area of phase persistence ε. Figure 22b–d show modified Lehrer plots of the T-Q system, which present, similarly to the Lehrer plot, the equilibrium between the phases of the Fe-N system and the nitriding mixture for ammonia (*a* = 1) and nitrogen-diluted ammonia (*a* = 0.5) and (*a* = 0.1). The graphs indicate with points the hydrogen content of the nitriding atmosphere for successive temperatures of the nitriding processes. The graphs for the process temperatures indicate the equilibrium hydrogen contents of the nitriding atmosphere, which correspond to points on the Lehrer diagram at the same temperatures.

### 5.7. Properties of the Solution Zone of the Nitrided Layer

**Figure 23:**
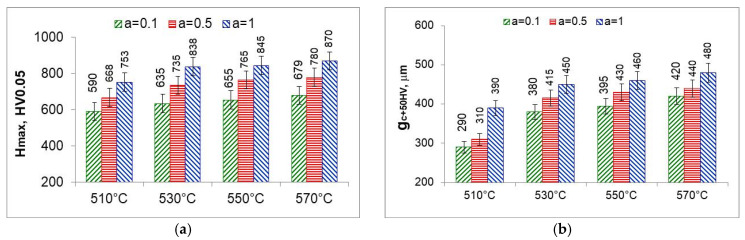
Maximum hardness (H_max_) (**a**) and effective thickness g_c50HV_ of the nitrided layer (**b**).

The solution zone of the nitrided layer was characterized by the maximum hardness (the highest hardness on the hardness distribution) (Figure 23a) and the effective thickness of the solution zone—core hardness plus 50 HV ($g_{C+50HV}$) (Figure 23b). The maximum hardness and effective thickness of the nitrided layers depend equally on the temperature and on the degree of nitrogen dilution of the ammonia atmosphere. Maximum hardness and effective thickness rise with rising temperature and rising ammonia content in the ingoing atmosphere *a*NH_3_*/b*N_2_ maximum values were obtained for the ammonia ingoing atmosphere of 100%NH_3_ (*a* = 1).

At 510 °C, the maximum hardness as well as the effective thickness of the solution layer are the lowest. Maximum hardness is the hardness immediately below the iron nitride layer, with the greatest degree of nitrogen dilution of the ingoing atmosphere. The saturation stage, on the other hand, is slowest due to the lowest availability of nitrogen; at the time of formation of the iron nitride layer, the saturation state of the substrate with nitrogen is much lower than the saturation state at temperatures above 530 °C and persists after the formation of the iron nitride layer. At temperatures above 530 °C, the degree of nitrogen dilution of the ingoing atmosphere has less effect on the maximum hardness and effective thickness of the nitrided layer. To obtain higher maximum hardness, one should use a single-component ammonia ingoing atmosphere while heating and for 30 min at process temperature and then change to a nitrogen-diluted atmosphere without changing the flow rate.

## 6. Control of the Nitriding Process in the NH_3_/N_2_ Atmosphere

Analyzing the results obtained in the NH_3_/N_2_ atmosphere with different volume percentages of ammonia and nitrogen, it can be concluded that nitrogen availability is an effective technological parameter for regulating the nitrogen flux from the nitriding atmosphere to the surface of nitrided steel while maintaining a constant value of nitrogen potential.

In the nitriding process, it is necessary to strive to adjust the nitrogen availability of the nitriding atmosphere to the mass of nitrogen that, at any given time, can be utilized by the nitrided substrate. The absorption capacity of the substrate decreases with nitriding time, according to the first derivative of the change in the mass of the nitrided layer as a function of time ($dm_{N-NL}(t)/dt$). The nitrogen mass of the nitrided layer ($dm_{N-NL}$) is the sum of the mass of nitrogen in the iron nitride layer ($m_{N-WL}$) and the mass of nitrogen in the solution zone $m_{N-SL}$):

(31) $$m_{N-NL}=m_{N-WL}+m_{N-SL}$$

The kinetics of nitrogen mass gain of the nitrided layer can be described in general by a power function ($k_{N-NL}\cdot t^{n})$ for the near-surface iron nitride layer as well as the solution layer:

(32) $$m_{N-NL}=k_{N-NL}\cdot t^{n}=\left(k_{N-WL}+k_{N-SL}\right)\cdot t^{n}$$

where: $k_{N-WL}$—constant of growth of iron nitride layer, $k_{N-SL}$—constant of power growth of solution layer, *t*—time, *n*—0.3–0.6.

As mentioned above, the absorption capacity of nitrided steel as a function of time is the derivative of the function (32) with respect to time:

(33) $$\frac{d\left(m_{N-NL}\right)}{dt}=\left(k_{N-WL}+k_{N-SL}\right)\cdot n\cdot t^{n-1}$$

To obtain a nitrided layer with the mass of $m_{N-NL}$, the availability of nitrogen must meet the condition:

(34) $$\beta \cdot m_{N_{2}}(t)\geq \frac{d\left(m_{NL}\right)}{dt}$$

where *β*—nitrogen utilization factor.

Relationship (35) enables to design of gas nitriding processes by defining the nitrogen availability of the nitriding atmosphere:

(35) $$\beta \cdot 0.625\cdot \frac{Q(t)}{1.5-Q(t)}\cdot F_{in}\geq \left(k_{WL}+k_{SL}\right)\cdot n\cdot t^{n-1}$$

where *F_in_*—flow rate of the ingoing atmosphere [dm^3^/min.].

The left side of Equation (35) will be modified depending on the process variant resulting from the type of ingoing atmosphere used and the available control parameters. In the case of single-component atmospheres, the availability of nitrogen can be changed by changing the flow rate of the ingoing atmosphere, but this is also accompanied by a change in the value of the nitrogen potential.

If the value of the nitrogen potential is to remain unchanged when changing nitrogen availability, it is necessary to use a two-component nitrogen ingoing atmosphere (*a*NH_3_*/b*N_2_). In such a case, inequality (35), after substituting Equation (14), will take the following form:

(36) $$\beta \cdot 0.625\cdot \frac{\left\{u\left(a(t)\;\right)\cdot {\left[N_{p}-v\left(a(t)\right)\right]}^{w\left(a(t)\right)}\right\}}{2-\left\{u\left(a(t)\;\right)\cdot {\left[N_{p}-v\left(a(t)\right)\right]}^{w\left(a\right)}\right\}}\cdot F_{i}\geq \left(k_{WL}+k_{sL}\right)\cdot n\cdot t^{n-1}$$

The proposed method of controlling and regulating the availability of nitrogen provides an alternative to current solutions for processes using NH_3_/N_2_ atmospheres.

## 7. Summary

As a result of analyzing the parameters of nitriding atmospheres obtained from an ammonia atmosphere and a two-component ammonia-nitrogen atmosphere, the parameters of the nitriding process were determined, which can be used to control the nitrogen flux from the nitriding atmosphere to the steel surface. As can be seen from Chapter 3, the universal control parameter in ammonia and ammonia-nitrogen nitriding processes is the hydrogen content of the nitriding atmosphere (Equations (10) and (14)).

Analysis of the error in the evaluation of nitrogen potential values as a function of ammonia content in the ingoing atmosphere indicates the need to improve the accuracy of process gas analyzers and flowmeters, which requires interaction with the entities that manufacture these devices.

Recording the change in the mass of nitrided samples and the nitrogen availability of the nitriding atmosphere made it possible to determine the degree of nitrogen utilization during nitriding (Figure 11 and Figure 12). The degree of nitrogen utilization decreases during nitriding as the absorption capacity of the nitrided steel decreases. To increase the nitrogen utilization rate, the availability of nitrogen should be reduced as the nitriding process progresses.

The results show that the degree of nitrogen dilution of ammonia makes it possible to effectively regulate the nitrogen concentration in the iron nitrogen layer without having to change the nitrogen potential value, as is the case when using ammonia atmosphere or ammonia with hydrogen or dissociated ammonia. Obtaining iron nitride layers in a wide temperature range (510–570 °C) with a structure of ε + γ’ and thicknesses up to 14 μm proves that the use of the NH_3_/N_2_ atmospheres can be used for nitriding machine parts and components for which nitriding is recommended. The advantage of the ε + γ’ layers is that they have high hardness and are not brittle, because the brittle ε phase in the iron nitride layer is in a mixture with the γ’ phase.

All analyzed samples show the presence of γ′-phase iron nitrides (Fe_4_N) and ε-phase iron nitrides (Fe_2–3_N), as confirmed by characteristic diffraction lines at 2θ = 40–58°.

Increasing the proportion of N_2_ in the ingoing atmosphere (lowering *a*NH_3_) leads to an increase in the proportion of the γ′ phase relative to the ε phase in the iron nitride layer. This is due to a reduction in nitrogen availability, which lowers the surface concentration of nitrogen.

The change in the proportion of γ′/ε phases correlates with the temperature of the process—as the temperature increases, a shift in phase equilibrium is observed according to the Lehrer diagram, and the γ′ phase content increases.

At 510 °C, with an atmosphere of 10%NH_3_/90%N_2_ (*a* = 0.1), a mixture of γ′-Fe_4_N and α-Fe or locally γ′+ε-Fe_2_-_3_N phases is observed, with the nitrogen concentration not exceeding 8.5% by weight. This limitation points to the key role of nitrogen availability as a limiting factor in the formation of richer nitride phases.

At higher temperatures (530–570 °C) and richer ammonia atmospheres (*a* = 0.5–1), the nitrogen content of the layer exceeds 9% by weight, leading to the formation of two-phase zones: ε at the surface and γ′ below.

Changes in the intensity of the diffraction lines confirm that the phase composition of the nitrided layer depends mainly on the combination of nitrogen availability and nitrogen potential, with the former parameter playing a key role when NH_3_/N_2_ atmospheres are used.

X-ray diffraction analysis showed the presence of iron nitride phases γ′ (Fe_4_N) and ε (Fe_2_-_3_N) in the near-surface layers of AISI 52100 steel nitrided in the NH_3_/N_2_ atmospheres. The identified phases correspond to stable Fe-N systems conforming to the Lehrer diagram, with nitrogen potentials achieved under the conditions studied.

Reducing the ammonia content of the ingoing atmosphere (*a*NH_3_ = 0.1) had the effect of reducing the nitrogen content of the near-surface layer to ≤8.5% by weight, which promoted the formation of layers with γ′ + α or γ′ + ε mixture structures. These layers were characterized by lower hardness and a limited thickness of the solution zone.

Increasing the NH_3_ content (*a* = 0.5–1.0) resulted in an increase in nitrogen content above 9.0% by weight in the near-surface zone of the layer, leading to dominance of the ε phase in the surface area and the formation of a bilayer structure (ε near the surface, γ′ below), as confirmed by both XRD and microscopic (SEM) observations.

Changes in the intensity of the diffractograms indicate an increase in the proportion of the γ′ phase as the dilution of the ingoing atmosphere with nitrogen increases, which is due to the reduced availability of nitrogen. This phenomenon correlates with the observed increase in the proportion of fine-crystalline γ′ structures in the SEM and the lower degree of porosity compared to ε-rich layers.

SEM observations showed the presence of layers with different phase morphologies: the layers with γ′ dominance were more homogeneous and less porous, while with ε dominance, there was a more developed porous zone, which may affect tribological properties and fatigue resistance.

The increase in process temperature (from 510 °C to 570 °C) resulted in an increase in the amount of γ′ phase at constant dilution, which can be linked to a decrease in the surface concentration of nitrogen at higher temperatures, according to the Fe-N equilibrium. This effect led to a higher proportion of γ′ at the expense of ε, despite identical atmospheric composition conditions.

## 8. Conclusions

1. Dilution of the NH_3_ atmosphere with nitrogen (N_2_) leads to a shift in equilibrium conditions toward the α-Fe phase, reducing the persistence of the γ′-Fe_4_N and ε-Fe_2_-_3_N phases.
2. Different NH_3_/N_2_ atmospheric compositions can lead to the same nitrogen potential value, due to the nature of the ammonia dissociation reaction and gas balance.
3. Dilution of the ammonia atmosphere with nitrogen is an effective tool for regulating the kinetics of growth of the nitrided layer and the thickness of the iron nitride layer.
4. With properly selected process parameters, the nitrogen availability of the nitriding atmosphere can be changed without significantly affecting the value of the nitrogen potential.
5. More than 90% of the mass of nitrogen absorbed by the nitrided layer is in the solution layer ($m_{N-NL}$), while only about 10% forms the layer of iron nitrides ($m_{N-WL}$).

## Figures and Tables

**Table 1:** Chemical composition (wt.%) of AISI 52100 steel.

Steel Grade	C	Si	Mn	S	P	Cr	Fe
AISI 52100	1.0	0.3	0.45	0.014	0.02	1.5	rest

**Table 2:** Nitriding process parameters.

T, °C	t, min	$N_{p}^{\varepsilon /\gamma \prime },{atm.}^{-0.5}$	*N_p_*, *atm.*^−0.5^	Inlet Atmosphere–*a*NH_3_/*b*N_2_			
				NH_3_, %	N_2_, %	*a*	*b*
510	300	1.9	15 ± 7%	100	0	1	0
			18 ± 12%	50	50	0.5	0.5
			20 ±30%	10	90	0.1	0.9
530	300	1.6	12.0 ± 8%	100	0	1	0
			14 ± 10%	50	50	0.5	0.5
			17 ± 30%	10	90	0.1	0.9
550		1.3	8.0 ± 6%	100	0	1	0
			10 ± 9%	50	50	0.5	0.5
			12 ± 30%	10	90	0.1	0.9
570	300	1.0	5.0 ± 5%	100	0	1	0
			6 ± 8%	50	50	0.5	0.5
			8 ± 30%	10	90	0.1	0.9

**Table 3:** Coefficients of approximation functions of the mass $m_{NL}(t)$ of samples as a function of nitriding time according to the parameters given in Table 2.

Atmosphere Composition		510 °C	530 °C	550 °C	570 °C
100% NH_3_ (*a* = 1)	$k_{NL}$	0.75	0.56	1.0	1.08
	n	0.35	0.45	0.37	0.38
50%NH_3_/50%N_2_ (*a* = 0.5)	$k_{NL}$	0.65	0.30	0.57	0.81
	n	0.34	0.53	0.44	041
10%NH_3_/90%N_2_ (*a* = 0.1)	$k_{NL}$	0.3	0.18	0.25	0.60
	n	0.44	0.60	0.56	0.44

## Data Availability

The original contributions presented in this study are included in the article. Further inquiries can be directed to the corresponding author.
